# Thermal noise-driven resonant sensors

**DOI:** 10.1038/s41378-024-00718-0

**Published:** 2024-06-26

**Authors:** Yan Qiao, Alaaeldin Elhady, Mohamed Arabi, Eihab Abdel-Rahman, Wenming Zhang

**Affiliations:** 1 School of Mechanical Engineering, Shanghai Jiao Tong University, Shanghai, China; 2 Department of Systems Design Engineering, University of Waterloo, Waterloo, ON Canada

**Keywords:** Electrical and electronic engineering, Sensors

## Abstract

MEMS/NEMS resonant sensors hold promise for minute mass and force sensing. However, one major challenge is that conventional externally driven sensors inevitably encounter undesired intrinsic noise, which imposes a fundamental limitation upon their signal-to-noise ratio (SNR) and, consequently, the resolution. Particularly, this restriction becomes increasingly pronounced as sensors shrink to the nanoscale. In this work, we propose a counterintuitive paradigm shift that turns intrinsic thermal noise from an impediment to a constituent of the sensor by harvesting it as the driving force, obviating the need for external actuation and realizing ‘noise-driven’ sensors. Those sensors employ the dynamically amplified response to thermal noise at resonances for stimulus detection. We demonstrate that lightly damped and highly compliant nano-structures with high aspect ratios are promising candidates for this class of sensors. To overcome the phase incoherence of the drive force, three noise-enabled quantitative sensing mechanisms are developed. We validated our sensor paradigm by experimental demonstrating noise-driven pressure and temperature sensors. Noise-driven sensors offer a new opportunity for delivering practical NEMS sensors that can function at room temperature and under ambient pressure, and a development that suggests a path to cheaper, simpler, and low-power-consumption sensors.

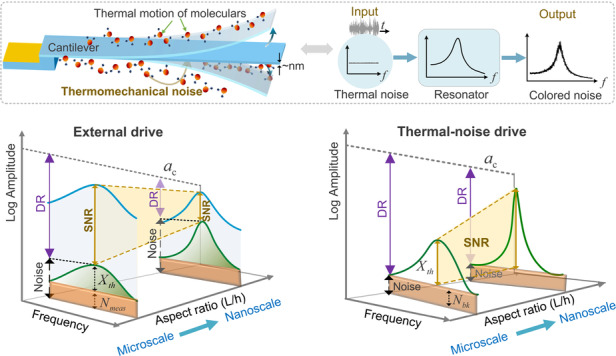

## Introduction

The last two decades have seen spectacular advancements and demonstrations of micro- and nano-scale electromechanical system (MEMS & NEMS) resonant sensors with the ability to detect the mass of single-molecule, weak atomic forces, and other ultra-small physical parameters^1,2^. For instance, state-of-the-art nanomechanical sensors have deployed nanoscale resonant wires, such as carbon nanotubes (CNTs)^3^, to achieve mass resolutions on the order of zepto-gram (10^-21^ gram)^4,5^ to yocta-gram (10^-24^ gram)^6^ and force sensitivity on the order of zepto-Newton (10^-21^ N)^7,8^. Such high-resolution sensing capabilities hold promise for detecting the mass of individual protons and the forces of individual nuclear spins^7^.

They achieve these extraordinary feats because of their ultra-small mass and high resonant frequencies^9^. However, a notable challenge in miniaturization is the increased impact of noise processes on these devices^10,11^. On the one hand, nanomechanical resonators are highly compliant rendering them susceptible to intrinsic and extrinsic disturbances. On the other hand, their motions are typically transduced into electrical signals proportional to the area of the sensing element, narrow nano-wires and ribbons, resulting in extremely small output signals, that can be easily overwhelmed by the noise floor^8^. Low signal-to-noise ratio (SNR) imposes direct fundamental limits on the sensitivity of amplitude-modulated detection^12,13^ and indirect limits on frequency-shift-based detection by undermining frequency stability^10,14–16^.

The noise processes that affects MEMS and NEMS sensors include the thermomechanical noise generated by the internal loss mechanisms in the resonator, thermoelectrical (Nyquist-Johnson) noise in the excitation circuitry, adsorption-desorption noise, and thermal/temperature fluctuations^17–20^. The first two noise sources contribute to the sensor response because they act as additive or multiplicative force (drive) noise^21,22^. The last two noise sources result in frequency fluctuations by tuning the effective mass, material properties (density, elastic modulus), or dimensions of the sensor^17^. In addition to those process noise sources, measurement noise resulting from the measurement instruments and readout circuits introduces additive white background noise to the output signal^23^.

For higher frequency MEMS and NEMS resonant sensors, thermal noise is the dominant noise source. It drives vibrations around the resonant frequencies. While this behavior has been used for characterization of resonators^24^, it ultimately reduces the SNR and sets the sensitivity limit for externally (coherently) driven MEMS and NEMS sensors^25^.

Schemes to increase the SNR in nanomechanical sensors have focused on suppressing the thermal noise level by operating in near-vacuum pressure^26^ or at cryogenic temperatures (–268 ^∘^C or less)^8^. These conditions, however, are impractical for sensing in fluid media or ambient air, such as the detection of gases, chemicals, and biological organisms. Further, the equipment required to create those conditions are complex, cumbersome, and expensive, thus making those schemes difficult to adapt for a general class of sensors.

Another promising strategy to improve the SNR in mechanical linear sensors is to increase the external driving power and access the full dynamic range (DR)^27^. Recently, Roy et al.^28^ demonstrated that the SNR and DR of linear sensors can be improved by increasing damping since higher damping increases the power handling capacity of the sensor, by retarding multivaluedness, and reduces the intrinsic noise level at resonance. This approach provides a roadmap to high-performance sensors in air and liquid environments. However, it calls for high driving power and is limited by nonlinearities in the sensor^29,30^. This is especially true for highly complaint NEMS sensors, where their DR is severely limited due to a high thermal noise level combined with a strongly nonlinear response^31,32^.

In this work, we propose a new sensor paradigm that transforms thermal noise from an obstacle to an integral part of the sensor by harnessing it as the driving force. Resonant structures are deployed to ‘color’ the inherent thermal bath energy, thereby creating features that can be used for sensing. Under this design paradigm, the sensors are self-powered by the inherent thermal noise present in their environment, without the need for external excitation. This paradigm would mitigate the limitations imposed by thermal noise upon micro- and nanomechanical sensors, and also suggest a pathway to low-power-consumption sensors. We define the SNR, DR, and frequency stability of those sensors and develop three noise-enabled quantitative sensing mechanisms immune to phase incoherence. We also present an electrical transduction method for motion detection of noise-driven sensors. As proof-of-concept, we demonstrate four noise-driven MEMS pressure and temperature sensors.

## Results

### Design paradigm

Noise processes encountered by resonators can be broadly classified into:

- process noise: noise that drive the resonator.
- measurement (transduction) noise: noise that affect the measurement signal but are not seen by the resonator, such as Johnson noise in electronic measurement instruments.

The resonator responds to the various process noise sources according to their characteristic time constant:

- It acts as a low-pass filter to high-frequency noise, those with a much shorter time constant than its fundamental period (*f* → *∞*), such as quantization noise from digital-to-analog converters.
- It is driven by noise with a similar time constant to its fundamental period resulting in a resonant response (Lorentzian curve) around its natural frequency, such as thermal noise.
- Noise with a much longer time constant, such as 1/*f*^33^ and 1/*f*^2^^17^, create drift in the resonator response.

**Fig. 1:**
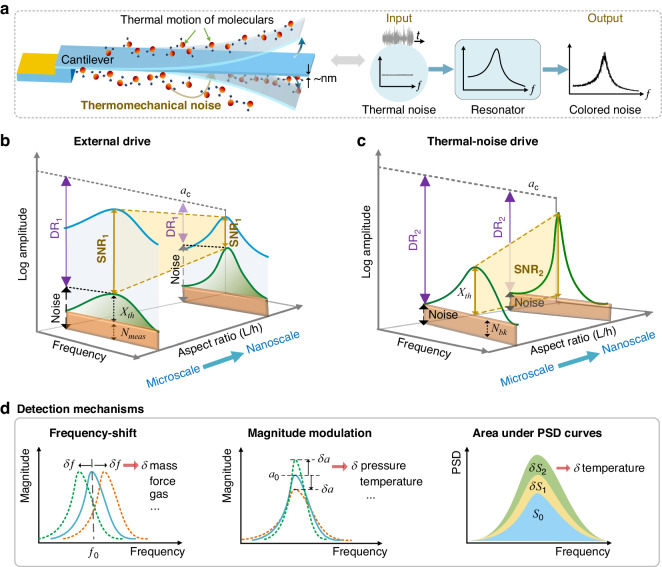
Design paradigm of the thermal noise-driven resonant sensors. **a** A schematic of the thermomechanical noise-driven cantilever and its driving mechanism. Comparison of the SNR and DR for (**b**) an externally driven sensor to that of (**c**) an intrinsically (thermal noise) driven sensor. The blue lines and green lines in (**b**) represent the sensor response (signal) under external force and its response to mechanical thermal noise *X*_*th*_, respectively. The background noise floor in (**b**) is the sum of *X*_*th*_ and measurement noise *N*_*meas*_, whereas in (**c**), the background noise is *N*_*bk*_, and *X*_*th*_ is sensor’s response signal. The length of the purple arrows in (**b**) and (**c**) represent the linear dynamic range of externally driven sensors (DR_1_) and thermal noise driven sensors (DR_2_), respectively. Similarly, the yellow arrows in (**b**) and (**c**) correspond to the SNR of externally driven (SNR_1_) and thermal noise driven (SNR_2_) sensors, respectively. **d** Three detection mechanisms for the noise-driven sensor: the resonant frequency-shift, the change in the resonant peak-magnitude and area under the power spectral density curve

Thermal noise is of particular interest since it is available throughout the frequency spectrum. It arises from molecular agitation, or random movement of particles within the device, its support structures, and the fluid media surrounding it^34–36^. Thermomechanical noise results in an unavoidable coupling of the thermal bath to the device and its motions even in the absence of any external excitation. The schematic of a thermomechanical noise-driven cantilever is presented in Fig. 1a (left). In those electromechanical systems where an electrical readout signal exists, the thermoelectrical noise generated by the function generator causes DC voltage fluctuations, thereby also contributing to the driving signal of noise-driven sensors^21^. In this work, we implement optical readout allowing us to restrict our interest to thermomechanical noise only.

#### Driving mechanism

Dissipative resonators in a thermal bath at a temperature above absolute zero (*T* > 0 K) experience inherent thermomechanical noise with a power spectral density (PSD) of^17^

1 $${S}_{th}(f)={S}_{0}=4{k}_{B}{T}\,{c}$$

where *k*_*B*_ is Boltzmann’s constant and *c* is the viscous damping coefficient. As per the equipartition theorem, the total thermal energy is harvested by the resonator and is transformed to its mean kinetic energy following the relationship^37,38^

2 $$\frac{1}{2}{m}_{e}{\omega }_{0 }^{2}\left\langle {x}^{2}\right\rangle =\frac{1}{2}{k}_{B}T$$

where angular natural frequency ${\omega_{0}}=2\pi {f_{0}},m_e$ is effective mass of the resonator, and $\left\langle {x}^{2}\right\rangle$ is the mean-square of its displacement. In the frequency domain, thermomechanical noise, initially manifesting white noise, becomes ‘colored’ when interacting with the resonator. This interaction results in an amplification of resonator’s output response at resonant frequencies, as shown in Fig. 1a (right). The power-based peak magnitude of displacement on-resonance, in units of length, can be found as

3 $${X}_{th}({f}_{0})=\sqrt{\frac{8{k}_{B}T\delta f}{k\mu }}$$

where *k* is the effective stiffness of the resonator and *μ* = *c*/*m*_*e*_ is the damping per unit mass. *δf* is the FFT (fast Fourier transform) linewidth. Details of Eq. (3) are in Supplementary S1.

Equation (3) shows that, under constant temperature, the peak magnitude of thermomechanical noise-driven sensors scales like $\propto 1/\sqrt{k\mu }$. This suggests the possibility of implementing a new class of mechanical systems that exploit intrinsic thermal noise as an actuation technique, without any driving force, to achieve extraordinary resonant vibration at ambient temperature, provided that we can build structures with ultralow stiffness (high compliance) and ultralow damping per unit mass. Cantilever-like structures are particularly advantageous for this purpose because they provide high compliance compared to their doubly-supported counterparts.

The SNR of traditional, externally driven, sensors compares the on-resonance signal *X*_*d*_($f_{0}$) in the presence of coherent external drive to background noise *X*_*N*_($f_{0}$) in its absence^26,28^

4 $${{{{\rm{SNR}}}}}_{1}=\frac{{X}_{d}({f}_{0})}{{X}_{N}({f}_{0})}$$

where *X*_*N*_($f_{0}$) is the sum in a power sense of the response to thermomechanical noise *X*_*th*_($f_{0}$) and measurement noise *N*_*meas*_($f_{0}$), ${X}_{N}({f}_{0 })=\sqrt{{{X}_{th}}^{2}({f}_{0 })+{{N }_{meas}}^{2}({f}_{0})}$ as shown in Fig. 1b. Expressed in decibel (dB), it takes the form as

$${{{{\rm{SNR}}}}}_{1}^{{{{\rm{dB}}}}}=20\,{\log }_{10}({{{{\rm{SNR}}}}}_{1})={X}_{d}^{{{{\rm{dB}}}}}({f}_{0 })-{X}_{N}^{{{{\rm{dB}}}}}({f}_{0 })$$

The contribution of thermomechanical noise to background noise often dominates that of measurement noise. For simplicity, we assume negligible measurement noise *N*_*meas*_ and use Eq. (3) to rewrite SNR_1_ as:

5 $${{{{\rm{SNR}}}}}_{1}\approx \frac{F}{\sqrt{8{m}_{e}\mu {k}_{B}T\delta f}}$$

where *F* is the amplitude of a harmonic external excitation. Details are in Supplementary S2.1.

In thermal noise-driven sensors, the drive force is intrinsic made of thermomechanical noise. The power level of this force is a known function of temperature. In contract to externally driven sensors which use phase angle dependent detection schemes and are thus exposed to the uncertainty injected by thermal noise in the phase angle^20^, we don’t use phase angle in detection. Instead, we employ power-based detection metrics. The power-based response to the known level of thermal noise is the ‘signal’. In this case, thermal noise is no longer background noise but an integral part of the sensor’s operational mechanism. On the other hand, measurement noise *N*_*meas*_($f_{0}$) remains a source of uncertainty in our metric and, thereby, continues to be part of background noise. Another factor contributing to background noise is the fact that *X*_*th*_($f_{0}$) in Eq. (3) assumes an infinite observation time and therefore an uniform distribution of the drive force over the frequency domain with a constant PSD. However, what we observe in practice is a nonuniform distribution that approaches uniformity as the observation time increases. Therefore, limited observation time of thermomechanical noise inevitably injects fluctuations into the sensor response it excites. The sum in a power sense of those two factors represent background noise:${N}_{bk}({f}_{0 })=\sqrt{{{N}_{meas}}^{2}({f}_{0 })+{{\sigma }_{X}}^{2}({f}_{0 })}$, where *σ*_*X*_($f_{0}$) captures the total uncertainty in the response to thermomechanical noise and in measurement noise due to limited observation time. For a given observation time *τ*, *σ*_*X*_($f_{0}$) can be estimated as the Allan deviation of the peak magnitude. Therefore, we define SNR for noise-driven sensors as the ratio of the on-resonance response to thermomechanical noise X_*th*_($f_{0}$) to the the background noise *N*_*bk*_($f_{0}$), as shown in Fig. 1c,

6 $${{{{\rm{SNR}}}}}_{2}=\frac{{X}_{th}({f}_{0 })}{{N}_{bk}({f}_{0 })}$$

Uncertainty in the response magnitude *σ*_*X*_($f_{0}$) can be reduced by a longer observation time *τ*. On the other hand measurement noise is dominated by the thermoelectrical noise within measurement circuits, which is characterized by a PSD of ${S}_{N}^{elec}=4{k}_{B}{T}_{r}R$, then we can write:

7 $${{{{\rm{SNR}}}}}_{2}\approx \sqrt{\frac{T}{k\mu R{T}_{r}}}$$

where *T*_*r*_ is the temperature of readout circuits, which may not be the same as the ambient (sensor) temperature *T* and *R* is the readout circuits resistance. Details are in Supplementary S2.2.

Comparison of the SNR expressions for externally driven sensors (Eq. (5)) to noise-driven sensors (Eq. (7)), shows that the latter can be made independent of temperature by placing the sensor and readout circuits in the same thermal bath *T* = *T*_*r*_. Alternatively, the ambient temperature *T* can act as a driving force while the readout circuit placed at a lower temperature *T*_*r*_ < *T* can serve as a signal amplifier^7^.

For cantilever beam-type noise-driven sensors, the SNR_2_ can be further written as

8 $${{{{\rm{SNR}}}}}_{2}=\sqrt{\alpha \frac{T}{R{T}_{r}}\frac{\rho {L}^{3}}{E{h}^{2}}}$$

where *L*, *h*, *E* and *ρ* are the beam length, thickness, Young’s modulus, and density, respectively. The coefficient *α* depends on the dominant energy loss mechanism. For generic linear damping it is given by:

$$\alpha =\frac{4L}{c}$$

For the special case of linear viscous damping, relevant to small motions in air, it reduces to^39^ :

$$\alpha =\frac{1}{\eta }$$

where *η* is the viscosity coefficient of air. In the case of squeeze-film damping, it is given by^39,40^:

$$\alpha =\frac{24.76{d}^{3}}{\eta {b}^{3}}$$

where *b* and *d* are the channel width and depth, respectively (the beam width and separation distance between the beam and the substrate).

The response of linear sensors is limited by a critical amplitude *a*_*c*_ representing the maximum power handling capacity and corresponding to the onset of nonlinearity (Details are in Supplementary S3). As a result, the dynamic range (DR) available to the externally driven sensors is limited to^27^

9 $${{{{\rm{DR}}}}}_{1}^{{{{\rm{dB}}}}}=20\,{\log }_{10}\left(\frac{{a}_{c}}{{X}_{N}({f}_{0 })}\right)={a}_{c}^{{{{\rm{dB}}}}}-{X}_{N}^{{{{\rm{dB}}}}}({f}_{0 })$$

We define the DR for noise-driven sensors as the ratio of the critical amplitude *a*_*c*_ to background noise *N*_*bk*_($f_{0}$)

10 $${{{{\rm{DR}}}}}_{2}^{{{{\rm{dB}}}}}=20\,{\log }_{10}\left(\frac{{a}_{c}}{{N}_{bk}({f}_{0 })}\right)={a}_{c}^{{{{\rm{dB}}}}}-{N}_{bk}^{{{{\rm{dB}}}}}({f}_{0 })$$

Figure 1b, c illustrates these relationships. We note that the definitions of SNR_2_ and DR_2_ for thermal noise-driven sensors presented here is a universal framework which is also applicable to scenarios involving both thermomechanical and thermoelectrical driving forces in the presence of electrical readout circuits. The difference lies in the fact that *X*_*th*_($f_{0}$) in the latter case should represent the cumulative response to both thermomechanical noise and thermoelectrical noise, in a power sense.

Typically, as the size of the resonator decreases, its aspect ratio *L*/*h* tends to increase, rendering it more sensitive to thermal noise, thereby leading to an elevation in its response to thermal noise^23^. Consequently, while the SNR of externally driven linear sensors gradually drops due to the rise in background noise floor as per Eq. (4), as indicated by the shrinking trend of the yellow area in Fig. 1b, the SNR of noise-driven sensors increases owing to enhancement of the useful signal *X*_*th*_($f_{0}$) while the background noise *N*_*bk*_ remains constant, as indicated by the expanding trend of the yellow area in Fig. 1c. The argument here is along the same lines of that of Roy et al.^28^. It just proposes a different solution to the challenge they point out.

Additionally, as the sensor size scales down (aspect ratio increases), nonlinearities become particularly pronounced, resulting in a decrease in the critical amplitude *a*_*c*_^27,32^. Consequently, the dynamic range of externally driven sensors (DR_1_) shrinks because the critical amplitude drops and the background noise floor is raised, as depicted by the changes in the length of the purple arrows in Fig. 1b. The dynamic range of thermal noise-driven sensors (DR_2_) also shrinks because the critical amplitude drops as depicted by the changes in the length of the purple arrows in Fig. 1c. However, DR_2_ is inherently larger than DR_1_ for the same sensor size.

The expression for the SNR of cantilever-based noise-driven resonant sensors, Eq. (8), suggests that nanoscale structures, such as 1D and 2D materials^7,8,41^, are ideal candidates for this class of sensors since they provide high aspect ratios, contributing to an enhanced SNR_2_. On the other hand, the use of nanoscale materials in externally driven linear resonant sensors poses a challenge as the background noise increases with increasing aspect ratio, limiting their SNR_1_ and DR_1_. Finally, we note that noise-driven sensors can access the full dynamic range DR_2_ at room temperature by increasing their aspect ratio since the critical temperature, where *X*_*th*_($f_{0}$) = *a*_*c*_, scales with the sensor’s dimensions as *T* ∝ *h*^2^/*L*, assuming the presence of only geometric nonlinearities.

#### Sensing mechanisms

The driving force in thermal noise-driven resonant sensors is incoherent by definition. While the response has a coherence time on the order of *Q*/$f_{0}$, it lacks deterministic phase information, which precludes the use of traditional phase-locked and other time-domain detection mechanisms. Therefore, those sensors require the use of detection mechanisms that are independent of the phase angle. Frequency domain sensing mechanisms that exploit the dynamic amplification offered by resonance are particularly appropriate for this purpose, such as measuring the magnitude or frequency shift of a resonant peak or the area under the power spectral density curve.

Stimuli that modulate the stiffness (potential field) or mass of a sensor can be detected as a shift, Fig. 1d, in the frequency of the resonant peak. Mistry et al.^40^ used this mechanism to achieve remarkable sensitivity to humidity in a noise-driven microcantilever sensor with a thickness of *h* = 250 nm.

Stimuli that affect the temperature and, thus, the level of noise forcing can be measured as a quantitative change in the magnitude of the resonant peak of the frequency-response curve or the area under the power spectral density curve, as per Eq. (2). On the other hand, stimuli that affect the quality factor, such as pressure^21^, can be measured as a quantitative change in the magnitude of the resonant peak but not the area under the curve, Fig. 1d.

The stability of the frequency and magnitude of the resonant peak are key for the performance of noise-driven resonant sensors. Multiple noise sources contribute to their fluctuations. Assuming that other sources of fluctuation in the sensor response are negligible compared to thermomechanical noise, we evaluated the fluctuation in peak frequency by deriving a closed-form expression of the fractional Allan deviation in Eq. (14) of the Material and Methods section. Comparing the frequency fluctuation of noise-driven sensors to that of externally driven sensors^15,23,28^, we note that the latter is counter-proportional to the product (*Q* SNR_1_) while the former is counter-proportional to $\tiny{\sqrt{Q\,{\omega }_{0 }}}$ only. Since noise-driven sensors can not use the SNR to improve their frequency stability, they must employ long-term averaging and a high product (*Q* ${\omega_{0}}$) to enhance their frequency stability^42^. Nano-cantilever beams combining dissipation dilution and phononic bandgap are promising candidates for a high (*Q* ${\omega_{0}}$) product^43,44^. On the other hand, increasing the averaging(observation) time is limited by the presence of low-frequency noise sources at 1/*f*^2^^10^, such as temperature fluctuation and adsorption-desorption noise.

#### Electronic Implementation

The output (motion-induced) current of electrostatic MEMS and NEMS sensors can be used to implement their electrical readout circuits^45,46^. This is, however, predicated on the availability of a harmonic drive force and, therefore, can not be used directly for noise-driven sensors. Instead, we propose a variant of the method specially adapted for noise-driven resonant sensors. Like traditional motion-induced current, a small voltage, *V*_*DC*_ is introduced to bias the sensor resulting in an output current. The fundamental component of this current is free of parasitics and directly proportional to the motional magnitude of the resonator, as per Eq. (S15) in Supplementary S4, unlike the case for externally driven sensors whose current is dominated by uncontrollable parasitic and feed-through currents as can be seen in the first and second terms on the right hand-side of Eq. (S11). This drastically increases the dynamic range of motion-induced current, since the feed-through current due to parasitic capacitance is typically three orders of magnitude higher than that due to motional capacitance^45^. It also eliminates the need for the lock-in amplifier typically used in the readout circuits to bypass the feed-through currents.

The output current can be amplified using a low-noise current amplifier and analyzed in frequency domain using a spectrum analyzer to detect resonant-frequency shifts or peak-magnitude modulations. Alternatively, the current RMS can be measured in time-domain. Since only a small bias is needed, a photodetector current amplifier can act as a suitable detection interface for noise-driven sensors^47^.

### Experimental results

#### Pressure sensors

To validate the proposed sensor paradigm, we present experimental demonstrations of noise-driven pressure and temperature sensors. In the first case, two classes of pressure sensors were implemented using MEMS cantilever beams. The FFT spectra of each sensor were used to derive calibration curves relating quantitative changes in the resonant peak of the tip velocity to ambient pressure.

**Fig. 2:**
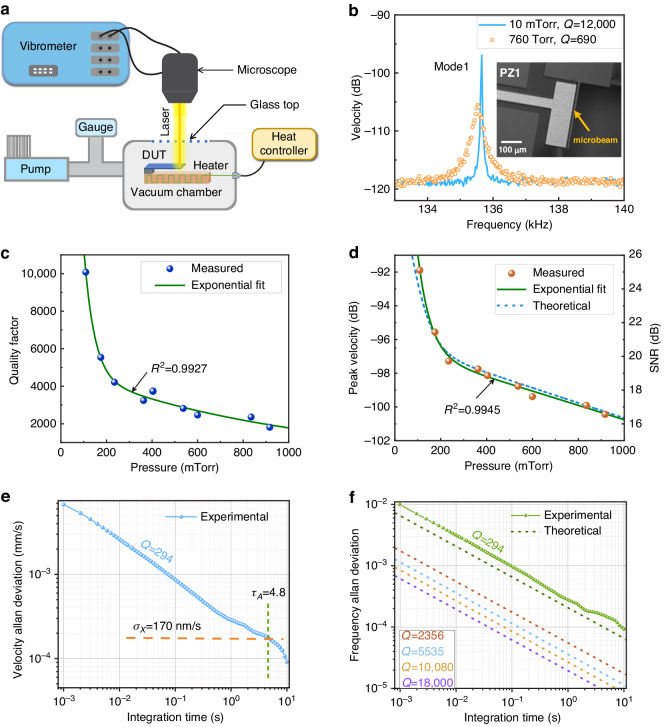
Noise-driven pressure sensors with high Q. **a** The experimental setup for testing of noise-driven resonant sensors. **b** Microscopic picture of PiezoMUMPS sensor PZ1 and its measured velocity spectrum in absence of external actuation. The measured (**c**) quality factor and (**d**) peak velocity response(left-hand ordinate) and SNR(right-hand ordinate) of sensor PZ1 as functions of pressure level. Two-term exponential fits of the measurements are shown in solid green lines. Theoretical predictions of the peak velocity are also shown in dashed blue lines. **e** The Allan deviation of the peak velocity. **f** The fractional Allan deviation of the resonant frequency

The experimental setup is shown in Fig. 2a. The device-under-test (DUT) was placed inside a vacuum chamber and a laser-Doppler vibrometer (Polytec Inc.’s MSV 400) was used to optically measure the tip velocity. The chamber pressure is varied by setting it initially to 7.5 mTorr, then allowing the pressure to increase slowly. As the pressure increases, the tip velocity is measured at intervals of one minute. Simultaneously, the ground truth pressure of the chamber is recorded independently using a commercial pressure sensor (CERAVAC CTR 100). Throughout the experiment, the sensor is maintained at room temperature (24 °C).

**Fig. 3:**
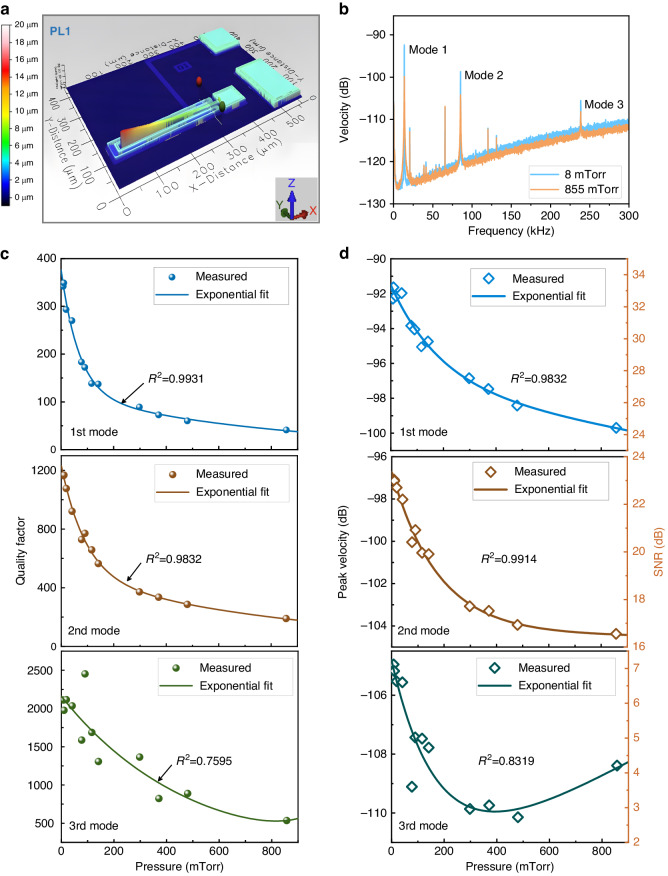
Noise-driven pressure sensors with low stiffness. **a** 3D profilometer picture of PolyMUMPS sensor PL1. **b** The measured velocity spectra of the first three bending modes. The measured (**c**) quality factor and (**d**) peak velocity and corresponding SNR of sensor PL1 at the first mode, second mode, and third mode as functions of pressure level. Two-term exponential fits of the quality factor and peak velocity are shown as solid lines

One set of pressure sensors (PZ1 and PZ2) was made of single crystal silicon cantilever beams fabricated in the PiezoMUMPs process, inset of Fig. 2b. The second set was made of polysilicon curved cantilever beam (PL1) fabricated in the PolyMUMPs process, Fig. 3a. The design dimensions of the sensors, their stiffness extracted from experimental measurements of the natural frequency, and their measured quality factor at 10 mTorr are listed in Table 1. The FFT was magnitude-averaged over 150 excitation cycles. The spectrum was post-processed to extract the peak magnitude and quality factor (via half-power bandwidth method) at resonance.

**Table 1:** Dimensions and properties of the sensors under test

Cantilever	Dimensions	Stiffness	Quality factor
	(*μ*m × *μ*m × *μ*m)	(N/m)	(10mTorr)
PZ1	315 × 10 × 10	18.5	18000
PZ2	320 × 10 × 10	16	15874
PL1	245 × 10 × 1.5	0.13	349
PL2	175 × 15 × 1.5	0.175	2800

The velocity spectra of the first bending mode of sensor PZ1 is shown in Fig. 2b. It compares the sensor’s response spectrum at low pressure, 10 mTorr to that at higher pressure, 760 Torr. The thermomechanical noise applied by the thermodynamic bath gives rise to resonant responses around the sensor’s natural frequency. It can be seen that the SNR of noise-driven sensors at low pressure is much higher than its counterpart at higher pressure due to the drop in dissipation *μ*, which is consistent with the expression of SNR_2_ presented in Eq. (7).

The measured quality factor and tip velocity at the resonance of the first mode for sensors PZ1 and PZ2 are shown in Figs. 2c, d and Figs. S1a, b, respectively, as pressure is varied. The quality factor decreases as the pressure level increases^48^, exhibiting a nonlinear one-to-one relationship, thereby resulting in a similar relationship between the peak velocity and pressure. The measured peak velocities in Fig. 2d were in the range of 9–25 μm/s, this is equivalent to an output current of 0.08–0.2 pA at a DC voltage of 40V. A two-term exponential fit of the measured quality factor, green lines in Figs. 2c and S1(a), results in *R*^2^ values of 0.9927 and 0.8699, respectively. The peak velocity measurements were also fitted to two-term exponential functions, shown in green solid lines in Figs. 2d and S1b. The *R*^2^ values of those fitting curves are 0.9945 for PZ1 and 0.9349 for PZ2. Those curves represent calibration curves of the pressure sensors.

Equation (3) was used to evaluate the peak velocity based on the quality factor fitting function. The theoretically predicted velocity, shown in blue dashed lines in Figs. 2d and S1b, are in close agreement with the experimental results, demonstrating the validity of the underlying analysis. On the other hand, it can be observed from the calibration curves that noise-driven pressure sensors are more sensitive to low pressure. This is expected given that the responsivity of the pressure sensor is proportional to its SNR, see Supplementary S5.1, Eq. (S19). Increasing the SNR further will allow the DR to extend to ambient pressure and beyond.

The fluctuations in peak magnitude and quality factor measurements are attributed to the limited observation time and measurement error. To examine that, we evaluated the stability of the magnitude and frequency of peak velocity of PZ1 for *Q* = 294 via Allan deviation as shown in Fig. 2e, f, respectively. The integration (response) time in the measurements shown in Fig. 2c, d was *τ*_*a*_ = 4.8 s, which corresponds to a peak velocity fluctuation of *σ*_*X*_($f_{0}$) = 170 nm/s, as per Fig. 2e. The right-hand ordinate of the Fig. 2d shows the SNR derived from the calibration curve as per Eq. (6), where the measurement noise floor for PZ1 was *N*_*meas*_($f_{0}$) = − 116 dB. The SNR is better than 15 dB throughout the pressure range of interest. The measured fractional frequency Allan deviation in Fig. 2f was found to be slightly larger than the predictions of Eq. (14), as the latter does not take into account the effect of measurement noise. The figure also shows the predicted fractional Allan deviation of frequency for higher quality factor sensors.

The velocity spectra of the first three bending modes of sensor PL1 are shown in Fig. 3b at pressures of 8 mTorr and 855 mTorr. The measured quality factor and peak velocity of sensor PL1’s first three modes of vibration are shown in Fig. 3c, d as functions of pressure, respectively. It can be seen that the peak magnitudes of the first two modes exhibit better sensitivity towards pressure and can serve as calibration curves for pressure detection. However, fluctuations in the quality factor of the third mode are significant with a lower *R*^2^ value. More significantly, the relationship between peak velocity and pressure, green symbols and line in Fig. 3d, is not single-valued.

The SNR, shown on the right-hand ordinate of the figures, drops as the mode number increases due to an increase in modal stiffness dominating a more limited variation in damping. For example, the damping per unit mass, measured at a pressure of 480 mTorr, was found to be *μ* = 1431, 1865 and 1683 for modes 1–3, respectively. In addition, a high SNR of approximately 30 dB is achieved for the first mode in vacuum.

These results indicate a limitation on the use of higher-order modes in noise-driven resonant sensors employing peak magnitude detection mechanism. It is a fundamental difference between this mechanism and the frequency-shift detection mechanism. In the latter case, responsivity and frequency stability improve with higher-order vibration modes^15^. as shown in Fig. S2. However, this limitation can be overcome by further increasing the sensor aspect ratio as per Eq. (8) and, consequently its SNR.

A comparison of the two classes of MEMS pressure sensors supports this conclusion. Although the damping of Piezo-MUMPs sensors is two orders of magnitude lower than that of the PolyMUMPs sensors, their SNR values are similar due to the countervailing effect of the lower stiffness of the PolyMUMPs sensors. An optimal nanoscale noise-driven sensor design would call for a combination of low damping and low stiffness, 1/*kμ*.

#### Temperature sensors

The same experimental setup was used to demonstrate noise-driven temperature sensors. The sensor under test (PL2) was fabricated in the Poly2 structural layer of the PolyMUMPs process. Its dimensions and properties are listed in Table 1. The DUT and a reference temperature sensor were placed side-by-side on top of a heating element (heater) within the vacuum chamber, Fig. 2a. A microcontroller was used to close the loop on the reference sensor signal and set the desired temperature. At each temperature setting, the system was left for 5 minutes to approach thermal equilibrium.

**Fig. 4:**
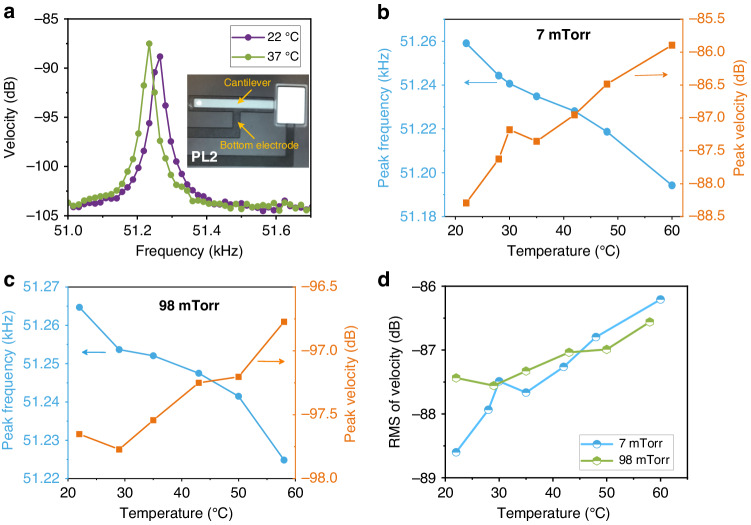
Noise-driven temperature sensors. **a** The averaged spectra of the tip velocity of thermal noise-driven sensor PL2 at two levels of temperature and a pressure of 7 mTorr. Measured peak frequency and magnitude of the velocity as functions of temperature under pressure levels of (**b**) 7 mTorr and (**c**) 98 mTorr. **d** Measured RMS of velocity as a function of temperature

The averaged spectra of the noise-driven sensor tip velocity are shown in Fig. 4a at temperatures of 22 °C (blue line) and 37 °C (orange line). The resonant frequency decreases as the temperature increases due to thermoelastic effects (changes in volume and elastic modulus) which tune the effective stiffness. Simultaneously, the peak magnitude grows. As the temperature changes from 22 °C to 37 °C, the ratio of change in the effective stiffness is *k*^22^/*k*^37^ = 1.001. The corresponding ratio of increase in peak velocity is *X*^37^/*X*^22^ = 1.163. This means that the dominant factor driving this increase in peak magnitude is the elevated level of thermomechanical noise, due to temperature rise, rather than the reduction in sensor stiffness.

The dependence of the resonant frequency and peak velocity on the temperature was investigated under two pressure levels: 7 mTorr and 98 mTorr. The quality factor in both cases is almost independent of the temperature, at *Q* ≈ 2750 for 7 mTorr and *Q* ≈ 245 for 98 mTorr. The results, Fig. 4b, c, show that the shifts in peak frequency and magnitude of velocity exhibit one-to-one relationships with temperature. While the former appears to be more robust than the latter, either of those metrics can serve as a calibration curve for a temperature sensor. The fluctuations in the magnitude of peak velocity are due to temperature drift and lower responsivity. As shown in Supplementary S5.2, Eq. (S20), the temperature responsivity is proportional to the SNR and counter-proportional to temperature. Similar to pressure sensors, the temperature responsivity of noise-driven sensors can be improved by increasing the sensor’s aspect ratio.

The measured RMS velocity, the square root of the area under the power spectral density curve of velocity evaluated as described in Supplementary S6, is shown as a function of temperature in Fig. 4d at pressure levels of 7 mTorr and 98 mTorr. It can be seen that the RMS of velocity is not sensitive to pressure, which is consistent with Eq. (2). This is the third calibration curve of the temperature sensor. In this case, the temperature responsivity can be further increased by reducing the effective mass of the cantilever as per Eq. (2).

## Discussion

In this paper, we present a new class of micro- and nanomechanical sensors, namely noise-driven sensors. Under this new design paradigm, no external actuation is required. Instead, the sensor is self-driven by intrinsic thermomechanical noise to resonance. In the presence of readout circuits, thermoelectrical (Johnson) noise also contributes to the drive signal. Highly compliant structures, such as nanoscale cantilevers endowed with high aspect ratios and high quality factors are ideal candidates for this class of sensors. This design paradigm suggests a new pathway for simpler and low-power-consumption nano-scale sensors operating at room temperature and under atmospheric pressure, promising to unleash NEMS sensors to realize their greater potential.

To characterize the thermal noise-driven sensor, we derived its SNR and DR and compared them to those of conventional sensors. It is found that the SNR of noise-driven sensors is counter-proportional to stiffness and damping per unit mass whereas that of conventional sensors is counter-proportional to total damping. This offers opportunities to enhance the SNR by increasing sensor’s aspect ratio. We also observed that the DR of noise-driven sensors is inherently larger than that of conventional sensors as the aspect ratio increases. These findings highlight significant advantages of noise-driven paradigm for nanoscale sensors.

Three noise-enabled quantitative sensing mechanisms immune to phase incoherence are developed. The first observes shifts in the resonant frequency in response to stimuli that modulate the structural stiffness or mass of the sensor. The second observes quantitative changes in the magnitude of the resonant peak in response to stimuli that affect the sensor damping and its excitation. The third measures the change of the area under the sensor power spectral density or the RMS of its motions to estimate the stimuli that impact the thermal noise level.

We validated the sensing mechanisms by demonstrating noise-driven pressure and temperature sensors. Calibration curves were created for pressure sensors based on the magnitude-shift of peak velocity and for temperature sensors based on the magnitude and frequency-shift of peak velocity and the RMS velocity. The analytical and experimental results indicate that the frequency stability of the resonant peak is proportional to the quality factor, resonant frequency and integration time. The magnitude stability was experimentally found to be proportional to the integration time. We note here a fundamental distinction between frequency-shift and magnitude modulation sensing mechanisms for noise-driven sensors. The former benefits from elevated operating resonant frequency and Q to enhance responsivity and frequency stability. As a result, higher-order modes are preferable for this mechanism, provided the SNR remains adequate (above 3 dB). Conversely, the latter mechanism requires a high SNR to improve the detection sensitivity, rendering high-order modes unsuitable due to their limited SNR.

The SNR of our sensors are enhanced by the low transduction noise level of optical vibrometry compared to that found in traditional electrical readout circuits. Moreover, recent advances have shown that on-chip optical detection^49^ is becoming practical. Further, we have previously shown that careful construction of electrical transduction circuits can produce superior SNR compared to optical transduction^50^.

Additional benefits of noise-driven resonant sensors encompass the reduction in background noise and the elimination of parasitics and feedthrough signals from the output, attributed to the absence of an actuation circuit. Moreover, the wide spectrum of thermal noise enables simultaneous excitation of vibrations in multiple higher-order modes, while direct excitation of these modes (beyond 1 MHz) has proven to be challenging. One possible challenge of noise-driven sensor lies in the need for longer integration time than the case for externally driven sensors to improve the stability of the measurands.

Although the sensor’s performance in current work is constrained by larger-sized (micro) beams and the relative insensitivity of silicon material properties to temperature, this marks an initial phase of exploring a new sensor technology paradigm and can serve as a foundational step for future research. The sensing performance of thermal noise-driven sensors will be improved through better sensor design (using nano-resonators with high aspect ratios), the use of more sensitive materials, and the integration of signal amplification. We plan to consider these approaches to unlock the greater potential of thermal noise-driven sensors in future work.

An alternative method to deploy the sensor paradigm proposed here is ‘noise aware’ sensors. In this case, the sensor accounts for a combined drive signal made of a coherent external force and intrinsic thermal noise. This approach would have better frequency stability than noise-driven sensors because it can exploit the rest of the dynamic range and thus increase the SNR. It would also have better SNR than traditional externally-driven sensors due to the reduction in background noise by leveraging mechanical-thermal noise as a driving force. On the other hand, the external drive signal will introduce feed-through noise and the including of incoherent noise in the drive signal will restrict detection methods to frequency domain methods, such as those discussed here. As a result, this approach may prove advantageous for micro-scale sensors but will have diminishing returns as sensors scale down as the available dynamic range beyond that occupied by the noise-driven response shrinks. We plan to investigate this approach in future work.

## Materials and methods

### 1. Allan deviation of the peak frequency

We describe the thermomechanical noise *ξ*_*th*_(*t*) as an additive stochastic force applied to the resonator. The resonators response can be described by the governing equation as

$${m}_{e}\ddot{x}+c\dot{x}+{\omega}_{0 }^{2}x={\xi }_{th}(t)$$

To characterize the stability of the peak frequency, we write the sensor displacement as

$$x(t,\tau )=a(\tau )\cos ({\omega}_{0 }t+\theta (\tau ))$$

where *a*(*τ*) is the displacement amplitude, *θ*(*τ*) is the phase deviation, and *τ* is a slow time-scale. Using the results of Qiao et al.^42^, we obtain approximate expressions for the slow evolution of the amplitude and phase as

11 $${a}^{{\prime}}=-\frac{c}{2{m}_{e}}a+\frac{{S}_{0 }}{4{m}_{e}^{2}{\omega }_{0}^{2}a}+\frac{1}{{m}_{e}}\sqrt{\frac{{S}_{0 }}{2{\omega}_{0 }}}{\psi }_{1}$$

12 $${\theta }^{{\prime} }=\frac{1}{{m}_{e}}\sqrt{\frac{{S}_{0 }}{2{\omega}_{0 }{a}^{2}}}{\psi }_{2}$$

where *ψ*_1_ and *ψ*_2_ are two independent unit white noise processes with zero-mean and the autocorrelation functions^51^

$${R}_{{\psi }_{i}}(\Delta t)=\frac{\delta (\Delta t)}{{\omega}_{0 }}\qquad ,i=1,2$$

To obtain the mean stationary response, we set

$$E({a}^{{\prime} })=0\quad ,\quad E({\theta }^{{\prime} })=0$$

and use Eq. (1) to obtain

13 $${a}^{2}=\frac{{S}_{0 }}{2{m}_{e}{\omega }_{0 }^{2}c}=\frac{2{k}_{B}T}{{m}_{e}{\omega }_{0 }^{2}}$$

which is consistent with Eq. (2).

Frequency fluctuation can be written as the rate change of the phase angle over the slow time

$$\delta \omega ={\theta }^{{\prime} }$$

Its spectral density can be obtained by taking the Fourier transform of the autocorrelation function of right-hand-side of Eq. (12) and using Eq. (13) to obtain

$${S}_{\delta \omega }(\omega )=\mu$$

We can estimate the frequency stability of the peak response over an averaging time *τ*_*A*_, by integrating the spectral density of frequency fluctuations over the resulting filter bandwidth^15^

$$\delta {\omega}_{0}({\tau }_{A})={\left(\int\nolimits_{{\omega}_{0}-\frac{1}{2}\Delta {\omega}_{A}}^{{\omega}_{0}+\frac{1}{2}\Delta {\omega}_{A}}{S}_{\delta\omega }(\omega )d{\omega} \right)}^{1/2}=\sqrt{\mu \Delta {\omega}_{A}}$$

where Δ*ω*_*A*_ = 2*π*/*τ*_*A*_ is the passband of the filter. Then the fractional Allan deviation of the resonator frequency due to thermomechanical noise is given by:

14 $${\sigma }_{A}^{th}({\tau }_{A})=\frac{\delta {\omega}_{0 }({\tau }_{A})}{{\omega}_{0 }}=\sqrt{\frac{2\pi }{Q{\omega}_{0 }{\tau }_{A}}}$$

### Supplementary information

Supplementary Material
