# Supplementary material for: Thermal noise-driven resonant sensors
Source: Microsyst Nanoeng. 2024 Jun 26;10:90. doi: 10.1038/s41378-024-00718-0 (PMC11208434; doi:10.1038/s41378-024-00718-0)
Supplement: Supplementary file 1 — Supplementary Material [file 41378_2024_718_MOESM1_ESM.pdf]

Supplementary Materials for

Thermal noise-driven resonant sensors

Yan Qiao[1], Alaaeldin Elhady[2], Mohamed Arabi[2], Eihab Abdel-Rahman[2,*], and Wen-Ming Zhang[1,*]

[1]*State Key Laboratory of Mechanical System and Vibration, School of Mechanical Engineering, Shanghai Jiao Tong University, Shanghai, China*

[2]*Department of Systems Design Engineering, University of Waterloo, Waterloo, ON, Canada*

[*]*Corresponding author:* wenmingz@sjtu.edu.cn (W. Zhang)
eihab@uwaterloo.ca (E. Abdel-Rahman)

Contents

S1. Peak magnitude of displacement

S2. SNR analysis

S3. Critical amplitude

S4. Motion-induced current in noise-driven sensors

S5. Responsivity analysis

S6. Calculation of RMS velocity

Figure S1. The measurement results of sensor PZ1 as functions of pressure level

Figure S2. Allan deviation of peak frequency for the first three mode of sensor PL1

S1. Peak magnitude of displacement

We describe the thermomechanical noise ξ_{th} as an additive stochastic force applied to the resonator. The resonator's response can be described by the governing equation as

$$m_e\ddot{x} + c\dot{x} + \omega_0^2 x = \xi_{th}(t)$$

The transfer function of a linear resonator relates the power spectral density (PSD) of the input thermomechanical noise to the PSD of the output sensor displacement by

$$S_x^{th}(f) = \frac{S_{\xi_{th}}(f)}{(k - 4\pi^2 f^2 m_e)^2 + (2\pi fc)^2} \tag{S1}$$

where $S_x^{th}(f)$ is in units of $\mu m^2 / Hz$ and $S_{\xi_{th}}(f)$ is in units of N^2 / Hz . k is the effective stiffness of the resonator and the mean-square of its displacement $\langle x^2 \rangle = \int_0^{+\infty} S_x^{th}(f)df$

Using Eq. (1) of the manuscript, The PSD of the displacement on-resonance ($f = f_0$) is

$$S_x^{th}(f_0) = \frac{4k_B T}{k\mu} \tag{S2}$$

where $\mu = c / m_e$ is the damping per unit mass. We note that,

$$\mu = \frac{2\pi f_0}{Q} = 2\pi\Delta f$$

where Q is the quality factor and Δf is the half-power bandwidth. The peak magnitude (in units of length) on-resonance can be found as

$$X_{th}(f_0) = \sqrt{S_x^{th}(f_0) 2\delta f} = \sqrt{\frac{8k_B T\delta f}{k\mu}} \tag{S3}$$

where δf is the FFT (fast Fourier transform) linewidth.

S2. SNR analysis

S2.1- SNR of externally driven sensors

The magnitude of displacement on-resonance $X_d(f_0)$ under a harmonic excitation force $F\cos(\omega t)$ is

$$X_d(f_0) = \frac{FQ}{k} \tag{S4}$$

where F is the amplitude of a harmonic external excitation.

The traditional definition of the signal-to-noise ratio (SNR), Eq. (4), for externally driven sensors is the ratio of the motional magnitude $X_d(f_0)$ under coherent external drive to background noise $X_N(f_0)$ on-resonance

$$SNR_1 = \frac{X_d(f_0)}{X_N(f_0)}$$

where the total background noise $X_N(f_0)$ is the sum of the response to thermomechanical noise $X_{th}(f_0)$ and measurement (instrumentation) noise $N_{meas}(f_0)$ in a power sense, $X_N(f_0) = \sqrt{X_{th}(f_0)^2 + N_{meas}(f_0)^2}$.

Assuming negligible measurement noise $N_m(f_0)$, we can use Eq. (S3) and (S4) to write SNR_1 as

$$SNR_1 = \frac{X_d(f_0)}{X_N(f_0)} \approx \frac{X_d(f_0)}{X_{th}(f_0)} = \frac{F}{\sqrt{8m_e \mu k_B T \delta f}} \tag{S5}$$

S2.2-SNR of thermal noise-driven sensors

We define the SNR for noise-driven sensors as the ratio of the on-resonance response $X_{th}(f_0)$ to thermomechanical noise to the uncertainty in this quantity $N_{bk}(f_0)$

$$SNR_2 = \frac{X_{th}(f_0)}{N_{bk}(f_0)} \tag{S6}$$

where $N_{bk}(f_0)$ is the sum in a power sense of the measurement (readout) noise $N_{meas}(f_0)$ and fluctuations $\sigma_X(f_0)$ (deviation): $N_{bk}(f_0) = \sqrt{N_{meas}{}^2(f_0) + \sigma_X{}^2(f_0)}$. The uncertainty in the response magnitude $\sigma_X(f_0)$ can be reduced by a longer observation time τ . Measurement noise $N_{meas}(f_0)$ includes the thermoelectrical (Johnson) noise of the measurement circuits, flicker ($1/f$) and shot noise in the photodetector, and discretization noise in the decoder. Among them, the thermoelectrical noise is dominant with a power spectral density of $S_N^{elec} = 4k_B T_r R$, where R is the total resistance of the readout circuits and T_r is their temperature. The magnitude (in units of length) of the noise floor due to thermoelectrical noise can be found as

$$N^{elec}(f_0) = \sqrt{8k_B T_r R \delta f} \tag{S7}$$

then we can write

$$SNR_2 \approx \frac{X_{th}(f_0)}{N^{elec}(f_0)} = \sqrt{\frac{T}{k\mu R T_r}} \tag{S8}$$

S3. Critical amplitude

The critical amplitude a_c represents the maximum amplitude up to which linear response can be assumed. Beyond that point is the onset of nonlinear response and multivaluedness. It is an inherent characteristic of the device and is primarily determined by the system quality factor Q, natural frequency ω_0, and the effective nonlinearity α. The equation governing this relationship can be derived as [1, 2]

$$a_c = 2\omega_0 \sqrt{\frac{1}{\sqrt{3} Q\alpha}} \tag{S9}$$

Eq. (S9) applies equally well to cantilevers, doubly-clamped beams or other oscillator geometries. Nonlinearity is an intrinsic characteristic in MEMS/NEMS resonators and originates from various sources, including geometrical nonlinearity, material nonlinearity, clamping defects, gradient forces, and tension, even in the absence of an external force. Additionally, some driving force like electrostatic force can also induce nonlinearity, potentially intensifying or mitigating system's nonlinear effects. Hence, while the degree of nonlinearity may differ between externally driven and thermally noise-driven devices, both share the same definition for critical amplitude outlined in Eq. (S9).

1. A. H. Nayfeh and D. T. Mook, Nonlinear oscillations. John Wiley & Sons, 2008.
2. Postma H W, Kozinsky I, Husain A, et al. Dynamic range of nanotube-and nanowire-based electromechanical systems[J]. Applied Physics Letters, 2005, 86 (22).

S4. Motion Induced Current

The total charge in a parallel-plate electrostatic resonator varies with the voltage $V(t)$ across it as

$$Q_c(t) = \left(C_p + \frac{\epsilon bh}{d - x(t)}\right) V(t) \tag{S10}$$

where b and h are the width and height of the plate / beam, respectively, d is the gap between them, ϵ is the free space permittivity, and C_p is the parasitic capacitance. The motion-induced output current of the resonator is the time derivative of the charge

$Q_c(t)$:

$$i = C_p\dot{V}(t) + \frac{\epsilon bh}{d - x(t)}\dot{V}(t) + \frac{\epsilon bhV(t)}{(d - x(t))^2}\dot{x}(t) \tag{S11}$$

In noise-driven sensors, voltage across the resonator is limited to a small bias voltage, $V(t) = V_{\mathrm{DC}}$ which reduces the output current to:

$$i = \frac{\epsilon bhV_{\mathrm{DC}}}{(d - x(t))^2}\dot{x}(t) \tag{S12}$$

And identically eliminate the parasitic current component, the first term of Eq. (S11).

Using Taylor series to expand the current up to the third order term and ignoring higher order terms yield:

$$i \approx C_0V_{\mathrm{DC}}\Big(\frac{1}{d} + \frac{2x(t)}{d^2} + \frac{3x(t)^2}{d^3}\Big)\dot{x}(t) \tag{S13}$$

where $C_0 = \dfrac{\epsilon bh}{d}$.

We assume that the resonator's response $x(t)$ to noise excitation is dominated by resonance frequency, such that:

$$x(t) = x_0 + x_1e^{j\omega t+\psi} \tag{S14}$$

where x_0 is a static deflection due to the DC bias, x_1 is the mean amplitude of motion, and ψ is a unit white noise process with zero-mean. The resulting current is:

$$i = j\gamma\Big(b_1x_1e^{j\omega t+\psi} + b_2x_1^2e^{2j\omega t+2\psi} + b_3x_1^3e^{3j\omega t+3\psi}\Big) \tag{S15}$$

where $\gamma = C_0V_{\mathrm{DC}}\omega$, $b_1 = (1 + 2x_0 + 3x_0^2)$, $b_2 = (2 + 6x_0)$, and $b_3 = 3$ are constants.

As shown in Eq. (S15), the output current is proportional to the motional magnitude of thermal noise driven resonators.

S5 Responsivity analysis

S5.1 - Responsivity of noise-driven pressure sensors

The responsivity of the noise-driven pressure sensors that employ peak magnitude detection can be expressed as

$$R = \frac{\partial X_{th}(f_0)}{\partial p} \tag{S16}$$

Using Eq. (S3), we write:

$$R = \left| \frac{\partial X_{th}(f_0)}{\partial Q} \cdot \frac{\partial Q}{\partial p} \right| = \left| \sqrt{\frac{k_B T \delta f}{Q k \omega_0}} \frac{\partial Q}{\partial p} \right| \tag{S17}$$

The measured quality factor Q was fitted to a two-term exponential function of pressure p of the form

$$Q(p) = A_1 e^{-b_1 p} + A_2 e^{-b_2 p} \tag{S18}$$

where A_1, A_2, b_1, and b_2 are positive fitting parameters. Substituting Eq. (S18) into Eq. (S17), we obtain:

$$\begin{aligned} R_p &= \left| -\sqrt{\frac{k_B T \delta f}{Q k \omega_0}} \left(A_1 b_1 e^{-b_1 p} + A_2 b_2 e^{-b_2 p} \right) \right| \\ &= \sqrt{\frac{k_B T \delta f}{Q k \omega_0}} b_1 \left(Q + A_2 (b_2 - b_1) e^{-b_2 p} \right) \\ &= \sqrt{\frac{k_B T \delta f}{k \mu}} b_1 + \sqrt{\frac{k_B T \delta f}{Q k \omega_0}} b_1 (b_2 - b_1) A_2 e^{-b_2 p} \\ &\propto \mathrm{SNR}_2 \end{aligned} \tag{S19}$$

This shows that pressure responsivity is proportional to the SNR.

S5.2 - Responsivity of noise-driven temperature sensors

Likewise, the responsivity of the noise-driven temperature sensors that employ peak magnitude detection can be derived from Eq. (S3) as follows:

$$R_t = \frac{\partial X_{th}(f_0)}{\partial T} = \sqrt{\frac{2 k_B \delta f}{k \mu T}} \propto \mathrm{SNR}_2 \tag{S20}$$

Therefore, the responsivity of temperature sensors is proportional to SNR and counter-proportional to temperature.

S6. Calculation of RMS velocity

Per Eq. (2), the thermal bath temperature can be measured as a quantitative change in the mean square (RMS) of velocity or displacement. In this work, we use the RMS of velocity as a metric. It can be measured either by time-domain averaging of velocity or by evaluating the square root of the area under the power spectral density curve of the velocity. Here, we adopted the latter course proceeding as follows

$$E[\dot{x}^2] = \int_{-\infty}^{+\infty} S_{\dot{x}}(f) df = S_{\dot{x}}(f_0) \frac{\pi}{2} \Delta f = \frac{(\dot{x}(f_0))^2}{2\delta f} \frac{\pi}{2} \frac{f_0}{Q} \tag{S21}$$

We measured f_0, $\dot{x}(f_0)$, and Q experimentally and evaluated the RMS of the velocity as:

$$RMS(\dot{x}) = \sqrt{E[\dot{x}^2]} \tag{S22}$$

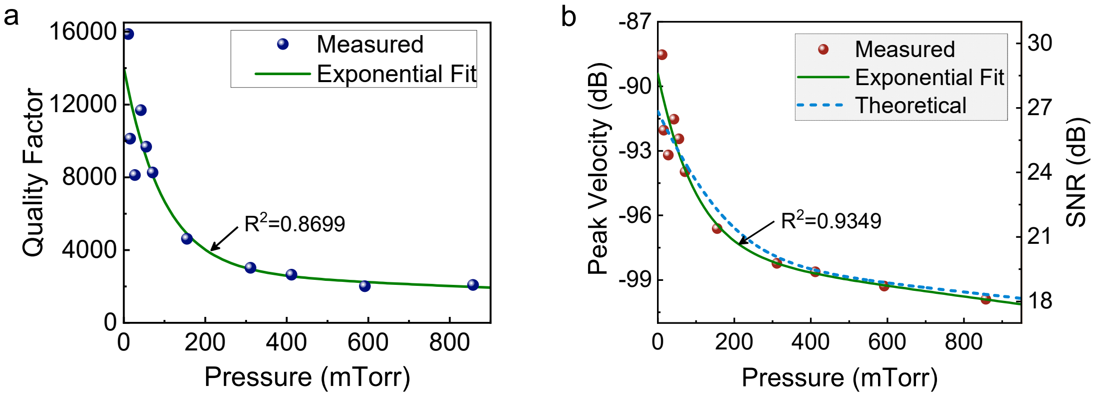

Figure S1: The measured (a) quality factor and (b) peak velocity response (left-hand ordinate) and SNR (right-hand ordinate) of sensor PZ2 as functions of pressure level. Two-term exponential fits of the measurements are shown in solid green lines. Theoretical predictions of the peak velocity are also shown in dashed blue lines.

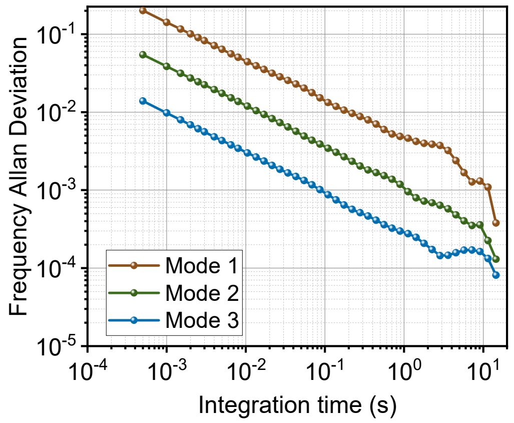

Figure S2: The fractional Allan deviation of the resonant frequency for noise-driven pressure sensors PL1 at its first three modes. The measurements were conducted at room temperature and under a vacuum of 10 mTorr.
